# CIDP/autoimmune nodopathies with nephropathy: a case series study

**DOI:** 10.1002/acn3.51754

**Published:** 2023-03-17

**Authors:** Yuwei Tang, Jing Liu, Feng Gao, Hongjun Hao, Zhirong Jia, Wei Zhang, Xin Shi, Wei Liang, Meng Yu, He Lv, Ying Tan, Zhiying Li, Yu Wang, Yun Yuan, Lingchao Meng, Zhaoxia Wang

**Affiliations:** ^1^ Department of Neurology Peking University First Hospital 8 Xishiku Street, Xicheng District Beijing 100034 China; ^2^ Department of Nephrology Peking University First Hospital 8 Xishiku Street, Xicheng District Beijing 100034 China; ^3^ Beijing Key Laboratory of Neurovascular Disease Discovery Beijing 100034 China

## Abstract

**Objective:**

The co‐morbidity of chronic inflammatory demyelinating polyradiculoneuropathy (CIDP)/autoimmune nodopathies with nephropathy has been gradually known in recent years. This study was intended to explore the clinical, serological and neuropathological features of seven patients with CIDP/autoimmune nodopathies and nephropathy.

**Methods:**

Among 83 CIDP patients, seven were identified with nephropathy. Their clinical, electrophysiological and laboratory examination data were collected. The nodal/paranodal antibodies were tested. The sural biopsies were performed in all the patients, and renal biopsies were operated in 6 patients.

**Results:**

Six patients had chronic onsets and one had an acute onset. Four patients exhibited peripheral neuropathy preceding nephropathy while two showed concurrent onset of neuropathy and nephropathy, and one started with nephropathy. All the patients showed demyelination in electrophysiological examination. Nerve biopsies showed mild to moderate mixed neuropathies including demyelinating and axonal changes in all patients. Renal biopsies showed membranous nephropathy in all 6 patients. Immunotherapy was effective in all patients, with two patients showing good response to corticosteroid treatment alone. Four of the patients were positive to anti‐CNTN1 antibody. Compared with anti‐CNTN1 antibody‐negative patients, antibody‐positive patients had a higher proportion of ataxia (3/4 vs. 1/3), autonomic dysfunction (3/4 vs. 1/3), less frequent antecedent infections (1/4 vs. 2/3), higher cerebrospinal fluid proteins (3.2 g/L vs. 1.69 g/L), more frequent conduction block on electrophysiological examination (3/4 vs. 1/3), higher myelinated nerve fiber density, and positive CNTN1 expression in the glomeruli of kidney tissues.

**Conclusion:**

Anti‐CNTN1 antibody was the most frequent antibody in this group of patients with CIDP/autoimmune nodopathies and nephropathy. Our study suggested that there might be some clinical and pathological differences between the antibody positive and negative patients.

## Introduction

Chronic inflammatory demyelinating polyradiculoneuropathy (CIDP) is an acquired immune‐mediated demyelinating neuropathy, which is classically characterized by symmetrical weakness and impaired sensation.^1^, ^2^, ^3^ Several cases of concomitant CIDP and nephropathy, related to either membranous nephropathy (MN) or focal segmental glomerulosclerosis (FSGS), have been reported.^4^, ^5^, ^6^, ^7^, ^8^ The concurrence of inflammatory neuropathy and nephropathy suggests common antigenic targets from the two tissues.

In recent years, antibodies targeted proteins of the nodal/paranodal regions, including neurofascin 155 (NF155), neurofascin 186 (NF186), contactin‐1 (CNTN1) and contactin‐associated protein 1 (Caspr1), have been reported in the cases of CIDP, which were also known as autoimmune nodopathies and showed specific clinical characteristics compared with the classical CIDP.^9^, ^10^, ^11^, ^12^ Recent studies suggested two distinct mechanisms of neuropathy comprised of macrophage‐induced demyelination in classical CIDP^13^ and paranodal axo‐glial detachment in patients with anti‐contactin‐1/neurofascin‐155 antibodies,^14^ indicating the unique pathogenesis of nodal/paranodal antibody‐positive CIDP. At the same time, the nodal/paranodal antibodies were also detected in some cases with concomitant CIDP and nephropathy,^15^, ^16^, ^17^, ^18^, ^19^, ^20^, ^21^, ^22^ which was why CIDP patients with nephropathy could be divided into antibody‐positive and antibody‐negative ones. However, due to the small number of reported related cases, whether the two types of patients had different clinical, pathological and prognostic features remained unclear. Here, using the new diagnostic criteria,^23^ we reported seven cases of CIDP/autoimmune nodopathies with nephropathy, and summarized their characteristics.

## Methods

### Patients

We reviewed the medical records of hospitalized patients in Peking University First Hospital between 2006 and 2021. From 83 cases, a total of 7 patients with CIDP and nephropathy were identified, whose clinical histories and results of examinations were collected, including laboratory finding, electrophysiological examination, nerve ultrasound and lumbosacral nerve root magnetic resonance imaging (MRI).

All patients met the diagnostic criteria of Joint Task Force of the European Academy of Neurology and the Peripheral Nerve Society.^23^ After the detection of antibodies against nodal/paranodal proteins, some patients were redefined as autoimmune nodopathies due to the positive results.

The nephropathy was defined as abnormalities of kidney structure or function, presented for >3 months. At least, one of the following abnormalities should be found: (a) Albuminuria (albumin excretion rate ≥ 30 mg/24 hs; albumin‐to‐creatinine ratio ≥ 30 mg/g); (b) Urine sediment abnormalities; (c) Electrolyte and other abnormalities due to tubular disorders; (d) Abnormalities detected by histology; (e) Structural abnormalities detected by imaging; (f) History of kidney transplantation; (g) glomerular filtration rate <60 mL/min/1.73 m^2^. All cases with nephropathies were confirmed by the consultation of nephrologists.

### Nerve conduction studies

Nerve conduction studies were performed with standard surface stimulation and recording techniques by a Keypoint4 electromyograph from Medtronic (Denmark). The patients were lying flat on the examination bed, and the skin temperatures were maintained 32–34°C in the limbs. The bilateral median, ulnar, radial, tibial, peroneal, and sural nerves were detected.

The parameters measured included the motor nerve conduction velocity (MCV), compound motor action potential (CMAP), distal motor latency (DL), F‐wave latency, sensory nerve conduction velocity (SCV), and sensory nerve action potential (SNAP). CMAP negative peak amplitude was measured from baseline to peak.

Motor conduction block was defined as ≥30% reduction of the proximal relative to distal negative peak CMAP amplitude, excluding the tibial nerve, and distal negative peak CMAP amplitude ≥20% of lower limit of normal values. And abnormal temporal dispersion is defined as >30% duration increase between the proximal and distal negative peak CMAP (at least 100% in the tibial nerve).

### Detection of nodal/paranodal antibodies

Sera from seven patients were collected and tested by cell‐based assay (CBA) for antibodies against nodal/paranodal proteins, including neurofascin 155 (NF155), neurofascin 186 (NF186) and contactin‐1 (CNTN1).

### Pathological examinations for nerve biopsy specimens

All the patients had sural nerve biopsies performed. Sural nerve biopsies were performed as described previously.^24^ The specimens were divided into two parts: one was fixed in 4% formalin before being embedded in paraffin and stained with hematoxylin and eosin (HE), luxol fast blue (LFB) and Congo red. The other was fixed in 3% glutaraldehyde and 1% osmium tetroxide and embedded in Epon‐812 after dehydration. Semithin sections were observed under light microscopy after toluidine blue staining while ultrathin sections were examined by electron microscopy after staining with lead and uranium.

The morphometric indices were assessed in toluidine blue‐stained semithin sections. Given the variability among individual fascicles, to evaluate the extent of myelinated nerve fiber loss objectively in each case, we manually counted the total number of myelinated fibers at least 3 fascicles on complete transverse sections of sural nerves on light microscopic images, captured at a magnification of 400×, covering the maximum nerve area. The total, the large (the diameter >7 μm), and the small (the diameter ≤7 μm) myelinated nerve fiber densities (MFDs) were calculated using Photoshop CC 2018 software (Adobe Systems).

### Pathological examinations for renal biopsy specimens

The renal biopsies were operated from all the patients except patient 6. Normal renal tissues were obtained from a patient with the renal cell carcinoma, undergoing nephrectomy. The renal tissues, far away from the site of carcinoma, were considered as the normal ones. Paraffin tissue was stained with hematoxylin and eosin (HE), periodic acid‐silver metheramine (PASM), periodic acid‐Schiff (PAS), and Masson trichrome. Immunofluorescence was performed on cryostat sections using a panel of FITC‐conjugated rabbit anti‐human antibodies to IgG, IgM, IgA, C3, C1q, and IgG1‐4 (Dako). Immunohistochemistry staining was performed using the goat anti‐CNTN1 antibody (Proteintech) and polink‐2 plus HRP anti‐goat DAB detection kit (GBI Labs).

### Ethics statement

All the subjects associated with this study signed informed consent, and this study was approved by the Ethics Committee of Peking University First Hospital.

## Results

### Clinical features of the patients

**Figure 1:**
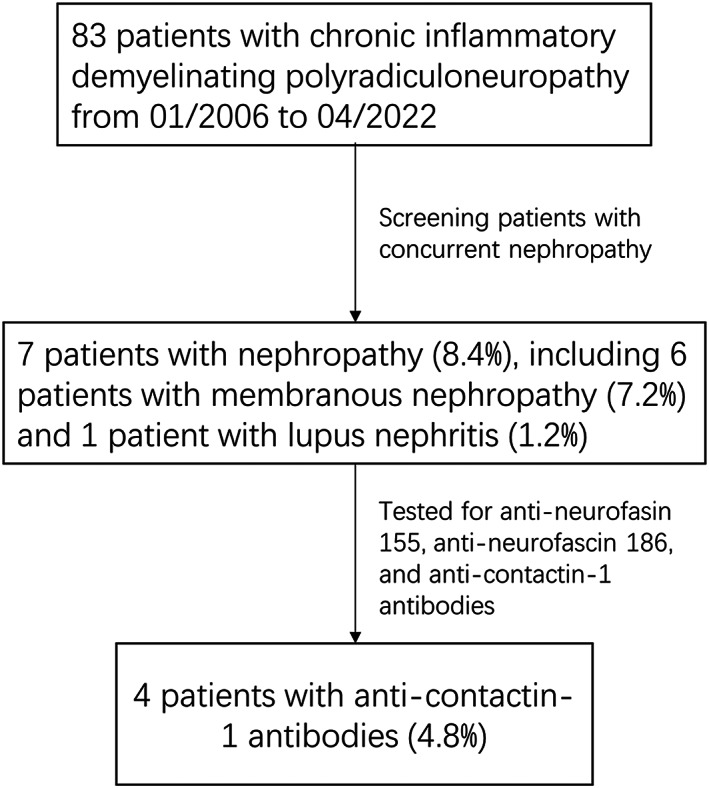
Flowchart.

We identified a total of seven patients (8.4%) with concomitant nephropathy among the 83 patients with CIDP hospitalized at our center from 2006 to 2022. Six of them were definitively diagnosed with membranous nephropathy by renal biopsy, and one patient did not receive renal biopsy due to mild renal lesions and was clinically considered lupus nephritis by consulting a nephrologist (Fig. 1).

The clinical features of the seven patients were listed in Table 1. Four of them were males. The average onset age was 44.7 years (ranging from 14 to 67). Three patients (patient 3, 4 and 7) developed prodromic infection, including fever, cough, sore throat and dermatitis of the right lower leg before onset. Only one patient (patient 3) was of the acute‐onset type, with a course of remission and relapse, while the others had chronic onset and a progressive course. As for the order of occurrence of the two diseases, four patients exhibited CIDP preceding nephropathy while two showed concurrent onset of CIDP and nephropathy, and one patient manifested with nephropathy first. All the patients showed limb weakness and/or numbness as initial symptoms. In patient‐ 2, 3 and 7, the initial symptoms ocurred only in lower limbs. Proximal limb weakness was more severe in three patients. Only one patient had muscular atrophy in thenar muscles of both hands. And no patient had cranial nerve involvement. Four patients (patient 1, 2, 3 and 5) showed signs of ataxia, such as positive Romberg's sign and instability in the heel–knee‐shin test. Only patient 5 showed obvious tremor. Three patients developed pain of lower extremities and four patients developed autonomic dysfunction, including anhidrosis, diarrhea and urinary incontinence. There were six patients showing abnormality of pinprick sensation, which was hypoesthesia in 4 and hyperalgesia in 2. Deep sensory examination showed reduced vibration sensation in the distal lower limbs in five patients.

**Table 1:** Clinical data of seven patients.

	Patient 1	Patient 2	Patient 3	Patient 4	Patient 5	Patient 6	Patient 7
Onset age (years)	60	46	14	57	67	33	36
Gender	M	F	M	F	M	F	M
Prodromic infection	−	−	+	+	−	−	+
Onset form	Chronic	Chronic	Acute	Chronic	Chronic	Chronic	Chronic
Initial symptom	Numbness of four limbs	Weakness of lower limbs	Weakness and numbness of lower limbs	Weakness and numbness of four limbs	Numbness of four limbs	Weakness and numbness of four limbs	Weakness and numbness of lower limbs
Course of disease	Progressive	Progressive	Remission and relapse	Progressive	Progressive	Progressive	Progressive
Sequence	Neuro→ renal	Neuro→ renal	Concurrent	Neuro→ renal	Neuro→ renal	Concurrent	Renal→Neuro
Weakness of limbs	UD > LD = LP=UP	LP > UD=UP>LD	LP > LD > UP=UD	LP > LD > UP=UD	UD = LD > UP = LP	LD > LP > UP>UD	LD > UD > UP = LP
Numbness	+	+	+	+	+	+	+
Muscle atrophy	−	−	−	−	−	−	+
Ataxia	+	+	+	−	+	−	−
Tremor	−	−	−	−	+	−	−
Pain of limbs	−	+	+	+	−	−	−
Autonomic symptoms	−	+	+	−	−	+	+
Pinprick sensation	Hypoesthesia	Normal	Hyperalgesia	Hyperalgesia	Hypoesthesia	Hypoesthesia	Hypoesthesia
Vibration sensation	Impairment	Normal	Impairment	Impairment	Normal	Impairment	Impairment
Autoantibodies against nodal/paranodal proteins	Positive CNTN1	Positive CNTN1	Positive CNTN1	−	−	Positive CNTN1	−
Autoimmune antibody	Positive ANA	Positive ANA	−	−	−	Positive ANA, dsDNA,	−
24 h‐UTP (g/24 h)	11.33	4	5.46	3.61	26	1.05	10.29
Serum creatinine (μmol/l)	84	54	42	60	143	78	904
CSF protein, g/L	ND	1.96	2.84	2.22	1.88	4.8	0.97
CSF cells per mm^3	ND	8	1	2	34	5	0
Albuminocytologic dissociation	ND	−	+	+	−	+	+
Nerve ultrasound	ND	ND	Normal	ND	ND	ND	Nerve thickening
Lumbosacral nerve root MRI	ND	ND	Normal	Normal	Normal	Nerve root thickening	Normal
Nerve biopsy	Axonal and demyelinating	Axonal and demyelinating	Axonal and demyelinating	Axonal and demyelinating	Axonal and demyelinating	Axonal and demyelinating	Axonal and demyelinating
Renal biopsy	MN stage 1	MN stage 1	MN stage 1	MN stage 1	MN stage 1–2	ND	MN stage 3
Treatment	GC + CTX	GC	GC	GC + CsA	GC + PE + RTX	IVIg+GC + CTX	PE + GC + CTX

> means more impaired muscle strength.

CsA, cyclosporine A; CSF, cerebrospinal fluid; CTX, cyclophosphamide; GC, glucocorticoid; LD, lower distal extremity; LP, lower proximal extremity; MN, membranous nephropathy; MRI, magnetic resonance imaging; ND, not detected; PE, plasma exchange; RTX, rituximab; UD, upper distal extremity; UP, upper proximal extremity; UTP, urine total protein.

Six patients presented with massive proteinuria, hypoproteinemia and hyperlipidemia, which were consistent with the diagnosis of nephrotic syndrome. They showed average 24 h‐urine total protein of 10.1 g/24 h (range from 3.61 to 26). Only patient 6 had a 24‐h urine protein of less than 3.5 g, which did not meet the diagnostic standard for nephrotic syndrome at this time. All patients tested negative to plasma anti‐PLA2R antibody, which was a specific antibody for membranous nephropathy. In the detection of autoimmune antibody, patient 1 and patient 2 revealed positive ANA (1:100). Patient 6 showed positive ANA (1:32,000) and anti‐dsDNA antibody (152 IU/mL, <100), leading to a concurrent diagnosis of systemic lupus erythematosus (SLE) with other related systemic involvement. All the patients, except patient 1, underwent lumbar puncture and albuminocytologic dissociation was present in four patients. Two patients had elevated leukocytes in cerebrospinal fluid (CSF). The average CSF protein in antibody‐positive patients was 3.2 g/L (1.96–4.80 g/L), higher than that in antibody negative patients (1.69 g/L, 0.97–2.22 g/L).

### Autoantibodies against nodal and paranodal proteins

**Figure 2:**
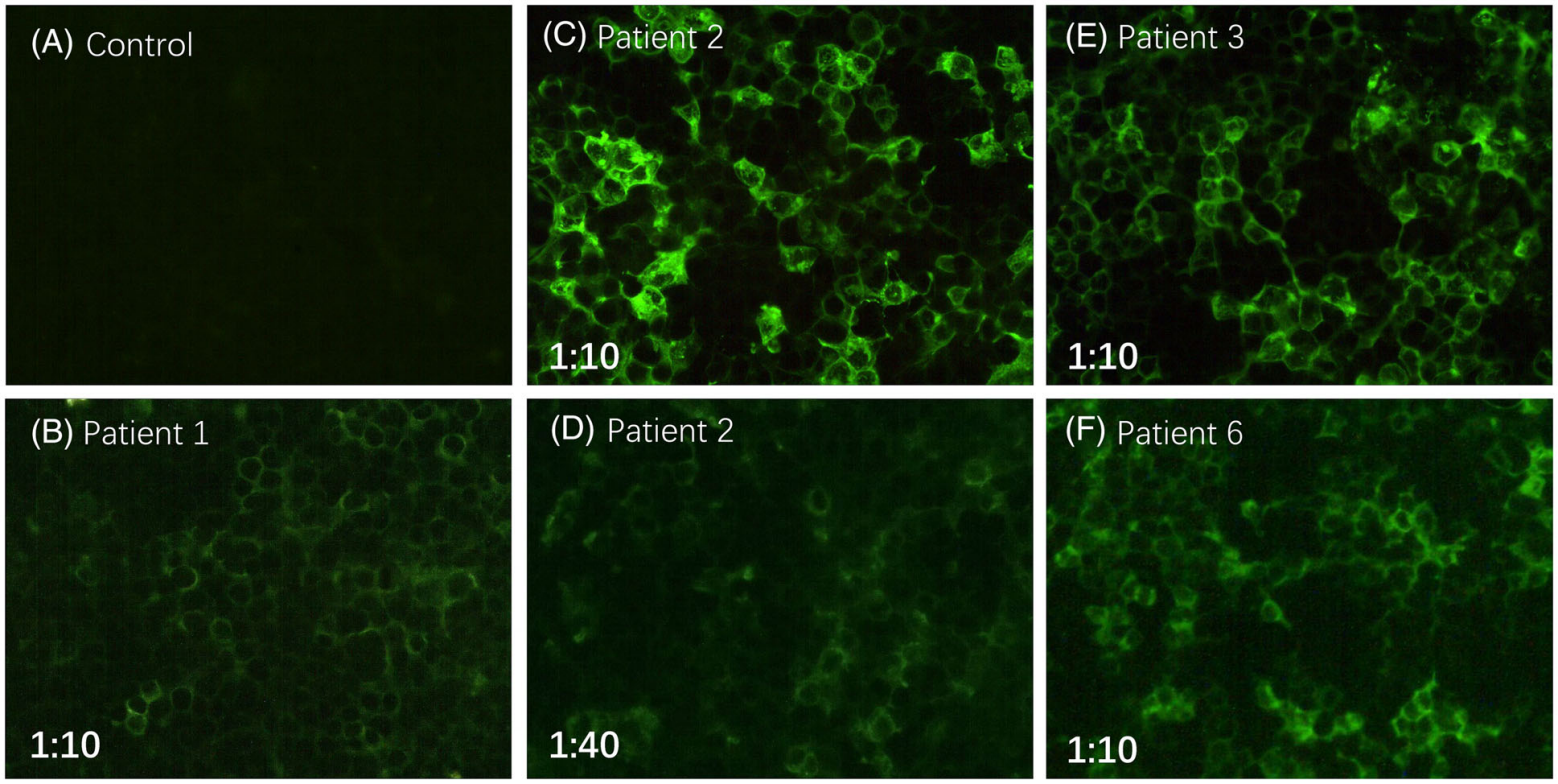
Immunofluorescence of contactin‐1‐transfected HEK293 cells with sera material of patient 1 (B), patient 2 (C, D), patient 3 (E), patient 6 (F) and control (A). Compared to negative control (A), the antibody was positive at titer 1:10 for patient 1, 3 and 6 (B, E, F), and positive with titer of 1:10 (C) and 1:40 (D) in patient 2.

The sera showed positive on cell‐based assay (CBA) to anti‐CNTN1 antibodies in four patients (patient 1, 2, 3, 6), while those of others were negative. Specifically, the antibody was positive at titer 1:10 in patient 1, 3 and 6, respectively, and positive with titer of 1:40 in patient 2 (Fig. 2).

### Nerve conduction studies

The results of nerve conduction studies (NCS) were shown in Tables 2 and 3. All the patients met the European Federation of Neurological Societies/Peripheral Nerve Society electrophysiological criteria for definite CIDP^23^. The prolonged distal latencies and decreased conduction velocities were detected in multiple motor nerves. And decreased amplitude of compound muscle action potential (CMAP) predominantly in lower limbs was also observed. In addition, patient 6 had severe motor nerve damage and most of the CMAPs were not elicited. Multiple motor conduction blocks were seen in four patients (patient 1, 3, 6, 7) with no temporal dispersion. Absent sensory nerve action potential (SNAP) in the upper and/or lower extremities was demonstrated significantly in six patients. For patient 3, sensory nerve conduction studies showed relatively normal sensory conduction velocities and amplitudes of SNAPs of upper and lower limbs.

**Table 2:** Electrophysiological examination of motor nerves in seven patients.

Motor	Patient 1			Patient 2			Patient 3			Patient 4			Patient 5			Patient 6			Patient 7		
	DL (ms)	CMAP (mV)	MCV	DL (ms)	CMAP (mV)	MCV	DL (ms)	CMAP (mV)	MCV	DL (ms)	CMAP (mV)	MCV	DL	CMAP	MCV	DL	CMAP	MCV	DL	CMAP	MCV
			(m/s)			(m/s)			(m/s)			(m/s)	(ms)	(mV)	(m/s)	(ms)	(mV)	(m/s)	(ms)	(mV)	(m/s)
L Median (<4, >5, >50)	**12**	**2.97**	**34**	**7.71**	12.4	52.7	**6.65**	9.1	52.6	**6.2**	8.3	46.9	**4.13**	7	50	**NR**			**9.21**	**4.4**	**18.6**
R Median (<4, >5, >50)	**11.4**	**3.346**	**27**	**8.96**	9.7	**36.8**	**6.27**	11.9	58.7	**15.2**	6.2	42.6	**4.52**	6.4	**46.3**	**6.93**	**3.6**	**24.1**	**8.77**	6.4	**17.9**
L Ulnar (<3, >4, >50)	ND			ND			ND			ND			**3.7**	6	**46.6**	**NR**			ND		
R Ulnar (<3, >4, >50)	ND			ND			ND			ND			**4.08**	6.7	**46.6**	**7.55**	**3.84**		ND		
L Peroneal (<5.3, >2, >40)	ND			**7.64**	**1.47**	59.9	**NR**			**7.8**	**0.9**	**26**	**8.03**	**0.93**	**26.3**	**NR**			**16.3**	**1.72**	**12.2**
R Peroneal (<5.3, >2, >40)	**17.6**	**0.443**	**19**	**6.56**	3.1	48.8	**11.6**	**0.3**	**36.8**	**6.2**	**0.3**	**35.3**	**8.03**	**0.63**	**30.7**	**NR**			**14.7**	**0.75**	**29.3**
L Tibial (<5, >3.5, >40)	**14.7**	**1.367**	**24**	ND			**7.29**	4.8	**36.1**	ND			**10.7**	**2.7**	**38.9**	**NR**			ND		
R Tibial (<5, >3.5, >40)	ND			ND			**9.53**	**2.1**	49.6	ND			**9.12**	**1.69**	**36.7**	**NR**			ND		

Abnormal values are indicated in bold.

CMAP, compound muscle action potential; DL, distal motor latency; MCV, motor nerve conduction velocity; ND, not detected; NR, no response.

**Table 3:** Electrophysiological examination of sensory nerves in seven patients.

Sensory	Patient 1		Patient 2		Patient 3		Patient 4		Patient 5		Patient 6		Patient 7	
	SNAP (μV)	SCV (m/s)	SNAP (μV)	SCV (m/s)	SNAP (μV)	SCV (m/s)	SNAP (μV)	SCV (m/s)	SNAP (μV)	SCV (m/s)	SNAP (μV)	SCV (m/s)	SNAP (μV)	SCV (m/s)
L Median (>5, >50)	**NR**		**NR**		33.8	58.4	**NR**		**NR**		**NR**		**NR**	
R Median (>5, >50)	**NR**		**NR**		9.1	**47.1**	**NR**		**NR**		**NR**		**NR**	
L Ulnar (>3, >50)	**NR**		**NR**		ND		**NR**		**NR**		**NR**		**NR**	
R Ulnar (>3, >50)	**NR**		**NR**		ND		**NR**		**NR**		**NR**		**NR**	
L Radial (>5, >50)	ND		9.7	**48.8**	ND		ND		**NR**		**NR**		**NR**	
R Radial (>5, >50)	ND		**3.9**	**39.9**	ND		ND		**NR**		**NR**		**NR**	
L Tibial (>1, >40)	ND		3.6	**34.9**	10.4	42.1	ND		**NR**		**NR**		**NR**	
R Tibial (>1, >40)	**NR**		ND		17	46.2	ND		**NR**		**NR**		**NR**	
L Sural (>1, >40)	3.474	44	ND		13.2	42.2	**NR**		**NR**		**NR**		**NR**	
R Sural (>1, >40)	1.219	**34**	3.9	**36.5**	12.5	43.1	**NR**		**NR**		**NR**		**NR**	

Abnormal values are indicated in bold.

ND, not detected; NR, no response; SCV, sensory nerve conduction velocity; SNAP, sensory nerve action potential.

### Nerve biopsy

**Figure 3:**
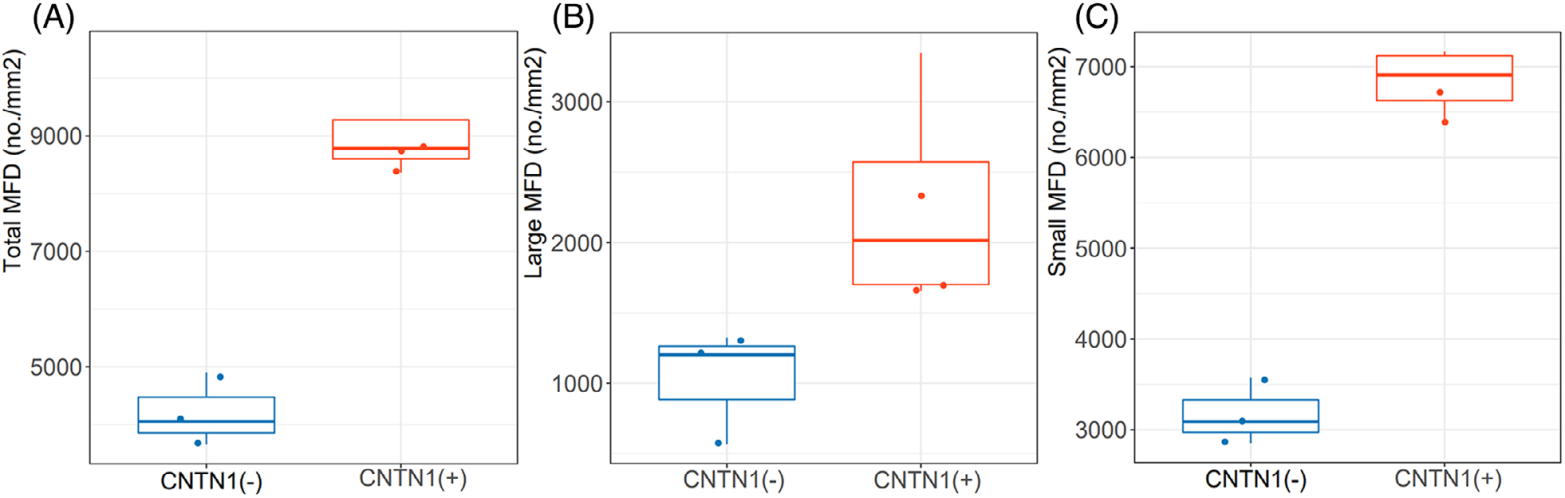
Difference in total MFD (A), large MFD (B) and small MFD (C) between anti‐CNTN1 antibody‐positive and anti‐CNTN1 antibody‐negative patients with combined nephropathy. Abbreviations: MFD, myelinated nerve fiber density;

**Figure 4:**
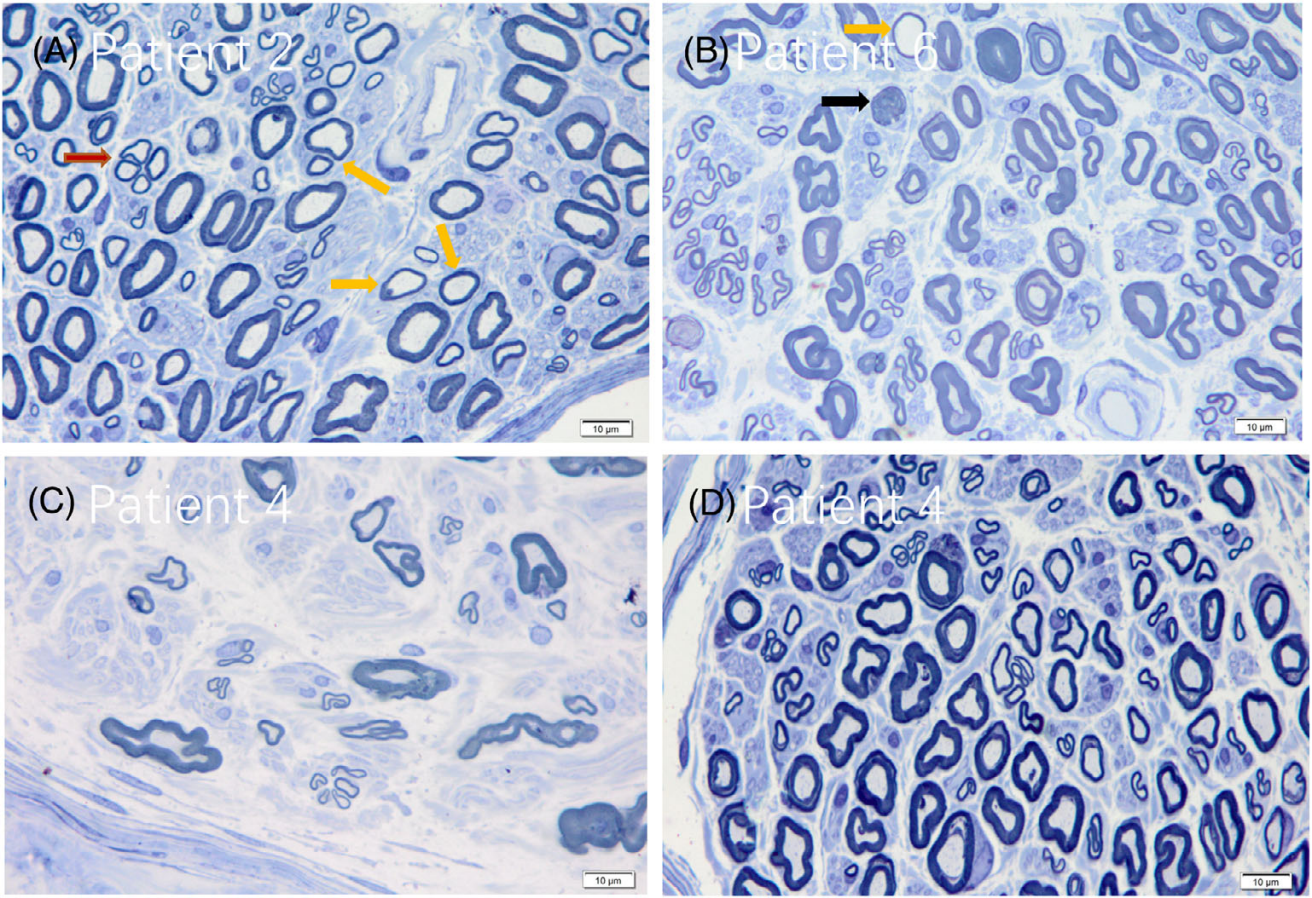
The semithin sections from patient 2 showed obvious axonal regeneration clusters (red arrow) and thin myelinated fibers (yellow arrow) (A). Patient 6 showed axonal degeneration (black arrow) and thin myelinated fibers (yellow arrow) (B). The loss degree of myelinated nerve fibers was different in different areas in patient 4 (C and D) (toluidine blue, x400).

The results of sural biopsies are shown in Table 4, Figures 3 and 4. All the patients revealed axonal injury including axonal degeneration and regeneration clusters. They all showed varying degrees of diminished myelinated fibers and the density of myelinated fibers was decreased mildly to moderately. In the semithin sections, only in patient 1, “onion bulb” formation was occasionally seen under the light microscope. Thin myelinated fibers could be seen in all patients, but the proportion of thin myelinated fibers in patient 2 was higher than that of others.

**Table 4:** Pathological features and MFD of seven patients.

Patient	Regeneration	Axon degeneration	Thin myelinated fiber	Onion bulb structure	Heterogeneous	Thickening of basement membrane	Small MFD (no./mm^2^)	Large MFD (no./mm^2^)	Total MFD (no./mm2)
1	−	+	+	+	−	+	6711.1	1654.5	8365.7
2	+	+	+	−	+	−	7104.3	3349.0	10,453.2
3	−	+	+	−	−	−	7168.6	1716.0	8884.5
4	+	+	+	−	−	+	2854.2	1201.8	4056.1
5	−	+	+	−	+	+	3573.1	1325.4	4898.4
6	−	+	+	−	−	−	6370.1	2313.6	8683.7
7	−	+	+	−	+	−	3091.7	567.8	3659.5

MFD, myelinated nerve fiber density.

There was thickening of capillary basement membrane in the specimens of three patients (patient 1, 4 and 5). In three patients (patient 2, 5 and 7), the degree of lesion between different nerve bundles was heterogeneous.

Specific myelinated nerve fiber density data were shown in Table 4 and Figure 3. The myelinated nerve fiber densities were higher in anti‐CNTN1 antibody‐positive patients than in anti‐CNTN1 antibody‐negative patients.

### Renal biopsy

**Figure 5:**
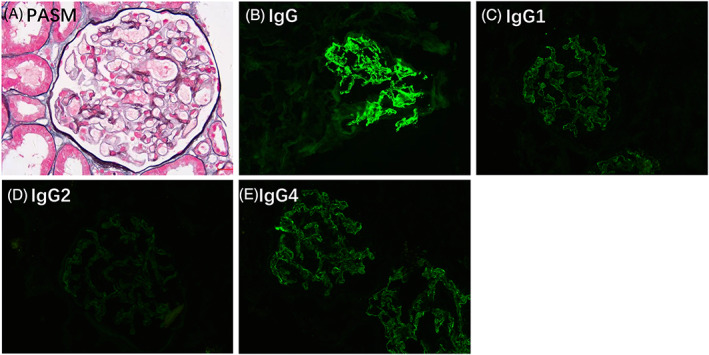
Renal pathological findings in the anti‐CNTN1 antibody‐positive patient (Patient 2). Light microscopy showed mild thickening of basement membrane (periodic acid‐silver methenamine, PASM, ×400). Immunofluorescence examination on frozen sections showed granular staining for IgG+++ (B), IgG1++ (C), IgG2+ (D) and IgG4++ − +++ (E) (×400).

**Figure 6:**
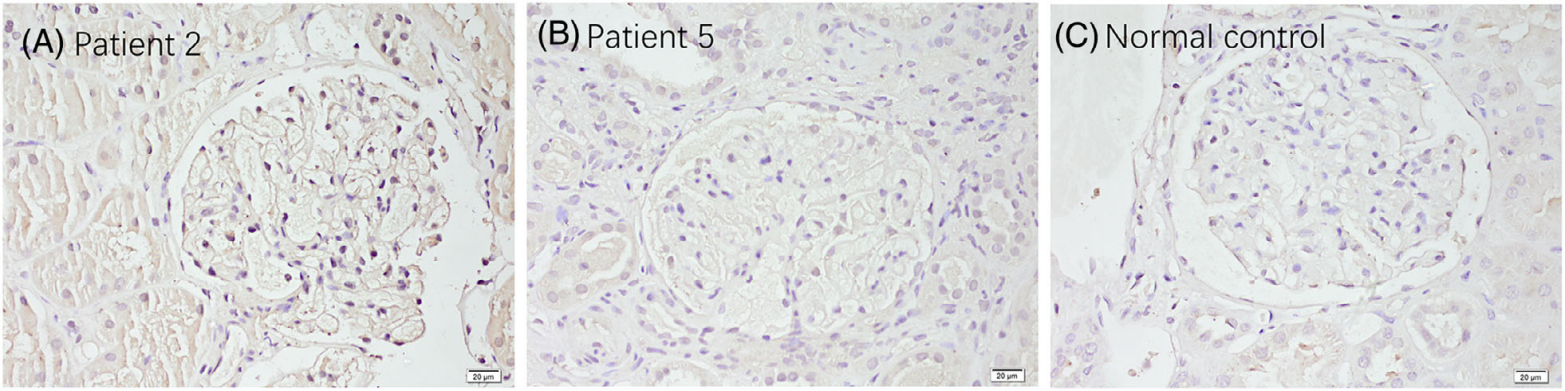
Immunochemistry staining for CNTN1 on deparaffinized sections. CNTN1 was expressed in the glomeruli from anti–CNTN1 antibody‐positive patients (A), but not in the antibody‐negative patients (B) or normal control (C) .

The renal pathological characteristics were detailed in Table 5. Except for patient 6, all patients underwent renal biopsy, which confirmed the diagnosis of membranous nephropathy. The average number of glomeruli sampled was 27 (range:14–43), with an average of 10% global sclerosis (range: 0–23%). Immunofluorescence staining showed deposition of IgG, IgM, IgA, C3, IgG1, IgG2, and IgG4, in glomerulus in different degrees (Fig. 5 and Table 5). We performed immunohistochemistry staining for CNTN1 on deparaffinized kidney tissues from anti–CNTN1 antibody‐positive patients, anti–CNTN1 antibody‐negative patients and normal control. We found that CNTN1 was expressed in the glomeruli from anti–CNTN1 antibody‐positive patients, but not in the antibody‐negative patients or normal control (Fig. 6).

**Table 5:** Pathological features of renal biopsy in 6 patients.

Patient	IgA	IgG	IgM	C3	C1q	IgG1	IgG2	IgG3	IgG4
1	−	+++	+	++	−	NR	NR	NR	NR
2	−	+++	−	+	−	++	+	−	++ − +++
3	−	+++ − ++++	−	+	−	++	−	−	++
4	++	+++	+	−	−	++	−	−	++
5	−	+++	+	++	−	++	+	−	−
7	−	+++	−	+	−	++	−	−	+++

NR, not recorded.

### Treatment

In terms of treatment, all the patients received corticosteroids therapy. Among them, patient 1, patient 2, patient 3 and patient 7 were given oral prednisone at doses of 50, 40, 80 and 5 mg/day respectively, while patient 4 and patient 5 were initially treated with intravenous methylprednisolone at doses of 20 and 40 mg/day, followed by oral prednisone. Patient 6 was given oral methylprednisolone 48 mg/day. In addition, cyclophosphamide (CTX) 50 mg twice daily was added after 1 week for patient 1. Patient 4 was also treated with cyclosporine A at doses of 50 mg twice a day. In addition, patient 5 was treated with plasma exchange for five courses and rituximab at a dose of 1 g. Patient 6 was treated with intravenous immunoglobulin (IVIg)，while hydroxychloroquine sulphate and CTX were added to the treatment as the patient also had SLE. Patient 7 was treated with plasma exchange seven times, followed by oral CTX 50 mg/day. Diuretic and neurotrophic drugs were used in all patients.

After treatment, the symptoms of all patients were improved in varying degrees, including decreased urinary protein, increased serum albumin, and improved muscle strength.

## Discussion

Several previous reports have described the association of CIDP with nephropathy.^6^, ^7^, ^8^, ^15^, ^16^, ^17^, ^18^, ^19^, ^20^, ^21^, ^22^, ^25^, ^26^, ^27^, ^28^, ^29^, ^30^, ^31^, ^32^, ^33^ In recent years, a variety of autoantibodies directing against the nodes/paranodes, including neurofascin 155 (NF155), contactin‐1 (CNTN1), contactin‐associated protein 1 (Caspr1), and neurofascin 186 (NF186) have been identified. Atay Vural et al.^11^ defined seropositive CIDP as patients with antibodies against neurofascin, CNTN1 and Caspr1 and suggested these subgroups of patients had unique characteristics that distinguished them from patients with seronegative CIDP. The new diagnostic criteria defined seropositive CIDP as the autoimmune nodopathies, not regarded as the CIDP variants,^23^ though in the previous literature, considered as the special subtypes of CIDP. Interestingly, thirteen of the patients with co‐morbid CIDP and membranous nephropathy were consistently found to be positive to anti‐CNTN1 antibodies,^15^, ^16^, ^17^, ^18^, ^19^, ^20^, ^21^, ^22^, ^33^ while two cases with anti‐NF186 antibodies were reported to present with acute‐onset CIDP and focal segmental glomerulosclerosis (FSGS).^34^ So pathological types of nephrotic syndrome might vary depending on the types of nodal/paranodal antibodies. However, more cases are needed to confirm this correlation.

Herein, we described co‐morbidity of CIDP/autoimmune nodopathies with nephropathy in seven patients, four of whom had positive anti‐CNTN1 antibody in the serum. All the pathological types of six patients who had a kidney biopsy were membranous nephropathy, and the only patient without the kidney biopsy had lupus nephritis, with a 24‐h urine protein level insufficient to justify the diagnosis of nephrotic syndrome. The co‐morbidity of CIDP with SLE was rare, with fewer than 20 cases reported in literature, from which a predominance of young women, especially nephritis and hematological involvement, was observed,^35^ but the anti‐CNTN1 antibody positive autoimmune nodopathy with lupus nephritis has never been reported before.

Our patients mainly had chronic sensory and motor neuropathies, without cranial nerve involvement. Most patients started with peripheral neuropathies, and only one patient had nephropathy first. None of the patients was positive to anti‐PLA2R antibody in plasma. A high proportion of these patients (4/7) had positive anti‐nodal/paranodal antibodies. And we found that the antibody‐positive patients were all positive to anti‐CNTN1 antibody, which strongly suggested that there was a close relationship between anti‐CNTN1 antibody and nephropathy. In four antibody‐positive patients, ataxia and autonomic dysfunction were more common, with a higher level of cerebrospinal fluid protein.

Literature review led to the finding that there were 13 anti‐CNTN1 antibody‐positive patients with MN previously reported. We found that their characteristics were relatively consistent: old onset age (average 60.2 years), male‐dominated (11/13), and most patients had peripheral neuropathy first or concurrent onset of neuropathy and MN. Unlike the reported anti‐CNTN1 antibody positive cases in literature, where the patients had an old age of onset, we identified for the first time a patient with an adolescent onset who started with a GBS‐like illness and a history of antecedent infection, indicating the wide age range of onset in the group of the disease.^35^ Interestingly, patient 5 showed tremor and ataxia with weakness and numbness mainly involving the distal limbs, which were consistent with the characteristics of anti‐NF155 or CNTN1 antibody positive cases, but the serum antibody test was negative, indicating the patient may have other unknown antibodies, which led to similar clinical manifestations with other antibody‐positive patients.

The NCS for all the patients met the European Academy of Neurology/Peripheral Nerve Society electrophysiological criteria for definite CIDP, which showed the evidence of demyelination.^23^ Previous literature reported that nerve biopsy from anti‐CNTN1 antibody‐positive patients showed axonal damage without the typical changes of demyelinating neuropathy such as thin myelinated fibers or onion bulbs, and no inflammatory cell infiltrates were seen.^12^, ^31^ Skin biopsy in these patients revealed elongated nodes of Ranvier,^22^, ^33^ adding to the evidence for nodal/paranodal abnormalities. In our study, all the patients showed mainly axonal damage, and demyelination such as thin myelinated fibers and atypical onion bulbs was also detected whether the antibody was positive or not. In fact, the characteristic pathological feature of antibody positive cases was destruction of axo‐glial attachment observed from the electron microscopy,^12^ which was not proved due to the limited number of longitudinal specimens of sural nerves in our study. Surprisingly, we found that for our seven patients, antibody‐negative patients had lower total, small and large myelinated nerve fiber densities than antibody‐positive ones, suggesting the possibly more malignant mechanism for the antibody‐negative patients, which led to the more obvious loss of myelinated nerve fibers, though we still needed more cases to prove the conclusion.

For treatment, the majorities of patients with CIDP/autoimmune nodopathies combined with MN were initially effective for corticosteroids, PE and IVIg.^21^ Allour seven patients were initially treated with corticosteroids and five of them were treated with plasma exchange or other immunosuppressive therapy, all of which were effective. While previous literature suggested that anti‐CNTN1 antibody‐positive patients showed an aggressive disease course and poor response to corticosteroids and IVIg, we found that some patients responded to corticosteroid treatment alone.

The exact mechanism of CIDP/autoimmune nodopathies with nephropathy, especially for antibody negative cases, was still unclear. It was proposed that severe nephropathy might lead to a reduction in the production of erythropoietin, resulting in a loss of its neurotrophic effects.^30^ In the case reported by Nazarali Samina et al.,^19^ the renal biopsy showed extensive CNTN1 expression throughout the glomerulus, suggesting the possibility that direct immune attack on the kidney by anti‐CNTN1 antibodies was the direct cause of nephropathy. Combined with the skin and nerve biopsy findings, it is hypothesized that anti‐CNTN1 antibodies attack both the kidney and the nodes of Ranvier, thus causing co‐morbidity of the two systems. In the case reported by Qianhui Xu, a patient developed CIDP 3 years after the onset of nephropathy. The authors speculated that the pathogenesis was not molecular simulation, but rather due to persistent positive anti‐PLA2R antibodies that may have triggered epitope spreading, and even induced intermolecular spreading of neoepitopes, leading to CNTN1 exposure.^15^ Moglie Le Quintrec's study, however, provided the first evidence of a common antigenic target, CNTN1, for patients with both anti‐CNTN1 antibody positive autoimmune nodopathies and MN. Eluted IgG from kidney tissues of patients with serum anti‐CNTN1 antibodies recognized CNTN1, and was able to bind paranodal nerve tissue, thus demonstrating that these antibodies could target CNTN1 in both tissues.^18^ In our research, after we stained the deparaffinized kidney biopsies with CNTN1 antibody, we found that the antibody‐positive patients showed CNTN1 antigen staining in the glomeruli, while the antibody‐negative patients and normal control did not show positive staining, suggesting the antigen unmasking in anti‐CNTN1 antibody positive cases, though the mechanism remained unknown.^18^ For the antibody negative cases, we speculated that there may be other autoantibodies targeting the common antigens expressed in nerve and kidney tissues, though more evidence is needed to prove the hypothesis.

In conclusion, the co‐morbidity of CIDP/autoimmune nodopathies and nephropathy was not rare and anti‐CNTN1 antibodies could be found in a high proportion of the cases. MN was the main type of nephropathy. To some extent, there were clinical and pathological differences between the antibody positive and negative patients. The albuminuria and renal function should be monitored for patients with CIDP/autoimmune nodopathies, especially in anti‐CNTN1 antibody positive patients.

## Author Contributions

Yuwei Tang collected the clinical data, drafted the initial manuscript and wrote the final manuscript. Jing Liu, Zhirong Jia, Wei Zhang, Xin Shi, Wei Liang, Meng Yu analyzed the electrophysiological data. Feng Gao, Hongjun Hao, He Lv took part in the study design. Ying Tan, Zhiying Li, Yu Wang analyzed the nephropathy of all patients. Lingchao Meng, Yun Yuan and Zhaoxia Wang reviewed the data and revised the initial draft. All the authors read and approved the final manuscript.

## Funding Information

This study was supported by Beijing Municipal Natural Science Foundation (No. 7194323).

## Conflict of Interest

The authors declare no conflict of interest.
